# Impaired Expression of Membrane Type-2 and Type-3 Matrix Metalloproteinases in Endometriosis but Not in Adenomyosis

**DOI:** 10.3390/diagnostics12040779

**Published:** 2022-03-22

**Authors:** Jane B. Maoga, Muhammad A. Riaz, Agnes N. Mwaura, Georgios Scheiner-Bobis, Ezekiel Mecha, Charles O. A. Omwandho, Ivo Meinhold-Heerlein, Lutz Konrad

**Affiliations:** 1 Department of Gynecology and Obstetrics, Justus Liebig University, 35392 Giessen, Germany; janeykemunto@gmail.com (J.B.M.); itassadriaz@gmail.com (M.A.R.); njokimwaura8@gmail.com (A.N.M.); ivo.meinhold-heerlein@gyn.med.uni-giessen.de (I.M.-H.); 2 Institute of Veterinary-Physiology and Biochemistry, Justus Liebig University, 35392 Giessen, Germany; georgios.scheiner-bobis@vetmed.uni-giessen.de; 3 Department of Biochemistry, University of Nairobi, Nairobi 00100, Kenya; ezekiel_mecha@yahoo.com; 4 Reproductive Biochemistry, Kirinyaga University, Kirinyaga 10300, Kenya; charlesomwandho@gmail.com

**Keywords:** endometrium, endometriosis, adenomyosis, membrane type-2 matrix metalloproteinase, membrane type-3 matrix metalloproteinase

## Abstract

Matrix metalloproteinases (MMPs) play an important role in menstruation and endometriosis; however, the membrane-type matrix metalloproteinases (MT-MMPs) are not well studied in endometriosis and adenomyosis. We analyzed MT2-MMP (MMP15) and MT3-MMP (MMP16) in eutopic endometrium with and without endometriosis and with and without adenomyosis and ectopic endometrium of deep infiltrating endometriosis (DIE), peritoneal endometriosis (PE), and ovarian endometriosis (Ov) by immunohistochemistry. Preferential expression of both proteins was observed in the glandular and luminal epithelial cells of the eutopic endometrium of patients with and without endometriosis with a ~2.5-fold stronger expression of MT3-MMP compared to MT2-MMP. We did not observe any differences during menstrual cycling and in eutopic endometrium of patients with and without endometriosis. Similarly, eutopic endometrium and adenomyotic tissue with and without endometriosis showed similar protein levels of MT2-MMP and MT3-MMP. In contrast, MT2-MMP and MT3-MMP protein was decreased in ectopic compared to eutopic endometrium and adenomyosis. The similar expression of MT2-MMP and MT3-MMP in eutopic endometrium in patients with and without endometriosis in contrast to the impaired expression in ectopic endometrium suggests that alterations occur after and not before endometrial implantation possibly by distinct interactions with the different environments. The differential protein expression of MT2/3-MMP in adenomyosis compared to endometriosis might suggest a different pathogenesis pathway for the two diseases.

## 1. Introduction

Endometriosis is a gynecological disease characterized by the presence of endometrial tissue in extra-uterine locations, such as the ovaries, pelvic peritoneum, and rectovaginal septum [1]. The condition very rarely occurs in extra-pelvic locations, such as the liver, colon, and lungs. Irrespective of the differential localizations, the histological appearance of ectopic glands is highly similar to uterine eutopic endometrial glands [2]. Remarkably, ectopic and eutopic glands are rarely in phase [3]. Three distinct types of pelvic endometriosis have been classified depending on tissue localization, namely ovarian, deep infiltrating, and peritoneal endometriosis [4]. 

Despite the vast number of women affected by endometriosis, its pathogenesis remains unclear, and different theories have been proposed [5]. The most accepted theory is retrograde menstruation, in which some of the menstrual blood together with endometrial tissue flow through the fallopian tubes into the pelvic cavity, followed by adhesion and invasion into the peritoneum [6]. Although retrograde menstruation occurs in about 76–90% of women of the reproductive age [7,8], only 0.7–8.6% of women in the general population suffer from endometriosis [9]. This indicates that other factors could be contributing to the development of endometriosis [10,11]. 

Adenomyosis is histologically characterized by the presence of endometrial tissue within the myometrium; thus, it is also known as endometriosis interna [12]. The additional eutopic endometrial tissue in the myometrium results in an enlarged uterus, and the implants are often surrounded by hypertrophic and hyperplastic myometrium [13]. Adenomyosis is, similarly to endometriosis, a benign uterine disease affecting 24.4% of women of reproductive age and is associated with endometriosis in 35% of cases as found in a hospital center [14]. However, in the general population, it is lower and was estimated to be around 2% [15]. Early menarche, short menstrual cycles, increased body mass index (BMI), surgical tissue damage [16], and history of depression [17] are possible risk factors. Adenomyosis is, like endometriosis, an estrogen-dependent gynecological disorder causing pelvic pain, abnormal uterine bleeding, and infertility [12] but is asymptomatic in ~1/3 of cases [18]. Similar to endometriosis, the pathogenesis of adenomyosis is also still unclear [12], but currently, two mechanisms, namely invagination and metaplasia, have been proposed [16]; however, our recent data favor the invagination hypothesis [19].

Matrix metalloproteinases (MMPs) are a family of zinc-dependent endopeptidases capable of degrading the different components of the extracellular matrix and are involved in different physiological processes, such as cell proliferation, differentiation, angiogenesis, apoptosis, and cell migration [20]. Based on their domain structure and substrate specificity, MMPs are classified into different groups, namely collagenases (MMP1, 8, and 13), gelatinases (MMP2 and MMP9), matrilysins (MMP7 and 26), stromelysins (MMP3, 10, and 11), and MT1-6-MMPs (MMP14, 15, 16, 17, 24, and 25) [21]. Increased expression and activities of MMPs are associated with excessive extracellular matrix degradation, and their deactivation can lead to insufficient extracellular matrix remodeling, resulting often in different pathological medical conditions [21,22,23]. A delicate balance is maintained between the MMPs and tissue inhibitors of metalloproteinases (TIMPs) that regulate MMP activity [21,24]. 

Membrane-bound MMPs (MT1-6-MMP) on the cell surface are part of an interface between the extracellular environment and the intracellular compartment; they are modifiers of the immediate cellular microenvironment, which in turn modulates cellular functions [24]. Consequently, all MT-MMPs are able to degrade extracellular components and, with the exception of MT4-MMP, also activate proMMP2 on the cell surface. This is remarkably the only soluble, active MMP detected in tissues to date [24]. 

In the human endometrium, mRNA expression and/or protein localization of MT1-MMP [22,23,24,25,26,27], MT2-MMP [27,28,29], MT3-MMP [27,28], and MT5-MMP have been reported [30]. Furthermore, in ectopic endometrium expression and localization of MT1-MMP and MMP2 in ectopic endometrium was found to be only increased in the secretory but not in the proliferative phase compared to eutopic endometrium [25], whereas Londero et al. [26] reported an increased MT1-MMP and MMP2 protein presence in endometriosis compared to eutopic endometrium. MT5-MMP mRNA expression was increased in ectopic endometrium of peritoneal lesions compared to eutopic endometrium of healthy persons [30].

To the best of our knowledge, MT2/3-MMPs protein levels have never been analyzed in endometriosis and adenomyosis; thus, in the present study, we analyzed both proteins in healthy patients versus endometrium with endometriosis (EM-En), endometrium with adenomyosis (EM-Ad), adenomyosis (Ad), ovarian endometriosis (Ov), peritoneal endometriosis (PE), and deep infiltrating endometriosis (DIE).

## 2. Materials and Methods

### 2.1. Patients and Ethical Approval

This study was approved by the Ethics Committee of the Medical Faculty of Justus-Liebig-University, Giessen, Germany (registry number 95/09). The participants gave written informed consent. All specimens were obtained by hysterectomy mainly from patients suffering from pain and by laparoscopy in cases of endometriosis (Table 1 and Table 2). Because of the scarcity of material, it was not always possible to exactly use the same patient for both proteins; however, in the case of MT2-MMP, there was an overlap in 99 of 152 samples with MT3-MMP and in the case of MT3-MMP, 100 samples from 113 cases were also used for MT2-MMP. In addition, we also obtained two healthy fallopian tubes and placentas, which were used as positive controls. We analyzed tissues from four groups: healthy endometrium (ctrl), endometrium with endometriosis (EM-En), endometrium with adenomyosis (EM-Ad), and all tissues with adenomyosis (Ad). Patients with both endometriosis and adenomyosis were grouped together. Additionally, we analyzed samples from deep infiltrating (DIE), ovarian (Ov), and peritoneal endometriosis (PE) (Table 1 and Table 2). The intraoperative findings were classified according to the Revised American Society for Reproductive Medicine Score and ENZIAN score [31]. Dating of endometrial tissue was based on the dates of the last menstrual cycle reported by the patients and histological evaluation by the pathologist. Despite the fact that the classification of DIE is still unclear [32], we relied on MRI and ENZIAN score [31].

Specimens were fixed in Bouin’s solution and partly in formaldehyde for histological evaluation by the pathologist and then were embedded in paraffin. Histological evaluation of the specimens was performed after staining 5 μm sections with hematoxylin and eosin.

### 2.2. Immunohistochemical Analysis and Quantification 

Serial sections of 5 μm were used to ensure that the same lesions were examined. Immunohistochemistry of Bouin-fixed or formalin-fixed specimens was performed according to the procedure previously described [33]. The EnVision Plus system (DAKO, Hamburg, Germany) was used according to the manufacturer’s instructions. Briefly, antigen retrieval was performed with citrate buffer (pH 6.0, DAKO), and then, the jars containing the slides were put into a steamer (Braun, Multi Gourmet) at 100 °C for 20 min and cooled for 20 min. Primary antibodies against MT2-MMP (diluted 1:100, cat-no PA5-13184, Invitrogen, Waltham, MA, USA) or MT3-MMP (diluted 1:100, cat-no PA5-79680, Invitrogen) were added and incubated in a humidified chamber overnight at 4 °C. After washing with PBS, samples were incubated with the secondary antibody (cat-no KA002, DAKO) for 30 min at room temperature. Staining was visualized with diaminobenzidine (liquid DAB K3467, DAKO) and counterstaining was done using Meyer’s hematoxylin (Waldeck, Germany). After dehydration in ethanol, the slides were mounted with Eukitt. Negative controls for IHC were prepared by omission of the primary antibody. Digital images were obtained using Leica DM 2000/Leica MC170/Leica application suite LAS 4.9.0 (Leica, Wetzlar, Germany) and processed with Adobe photoshop CS6. Following staining, the staining intensity was quantified using the HSCORE (negative = 0, weak = 1, moderate = 2, and strong = 3). The score was calculated using the formula 3 × strongly stained (%) + 2 × moderately stained (%) + 1 × weakly stained cells (%), giving a range of 0 to 300. All glands or cysts were used for evaluation of the HSCORE. 

### 2.3. Statistics

Values are presented as mean ± standard error of the mean (SEM) or median ± standard deviation (SD). HSCORE values between the different groups were analyzed using one-way analysis of variance (ANOVA). Comparison between more than two groups was done using Kruskal–Wallis test. *p*-Values ≤ 0.05 were considered to be significant. Graphpad prism 6.01 (www.graphpad.com) was used for the statistics.

## 3. Results

**Figure 1:**
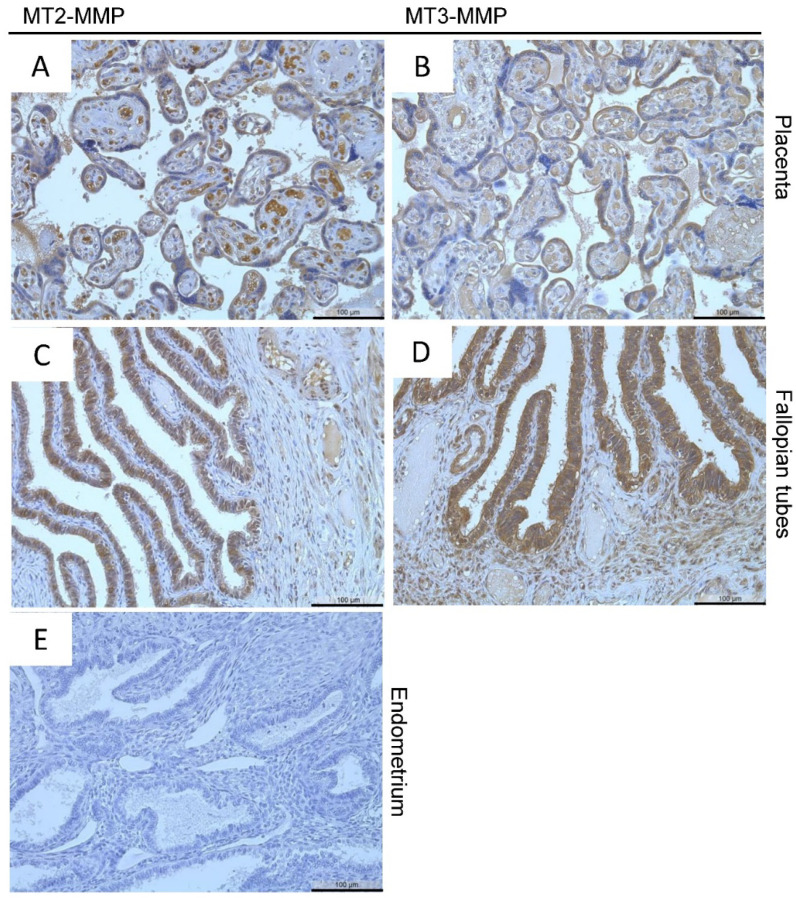
Immunohistochemical detection of MT2-MMP (**A**,**C**) in the placenta and of MT3-MMP (**B**,**D**) in the fallopian tubes (positive controls), No staining was found in the negative control. Counterstaining was performed with hematoxylin. Magnification (**A**–**E**) 20×, scale bars 100 µm.

From the proteinatlas database (www.proteinatlas.org) (accessed on 31 March 2020), we identified tissues from the female reproductive tract that express MT-MMPs and used them as positive controls: the placenta and the fallopian tubes. Both MT2-MMP and MT3-MMP are localized in the trophoblast cells of the placenta (Figure 1A,B) and in the glandular cells of the fallopian tubes (Figure 1C,D) comparable to localizations shown in the proteinatlas database. No staining was observed in the negative control (Figure 1E).

**Figure 2:**
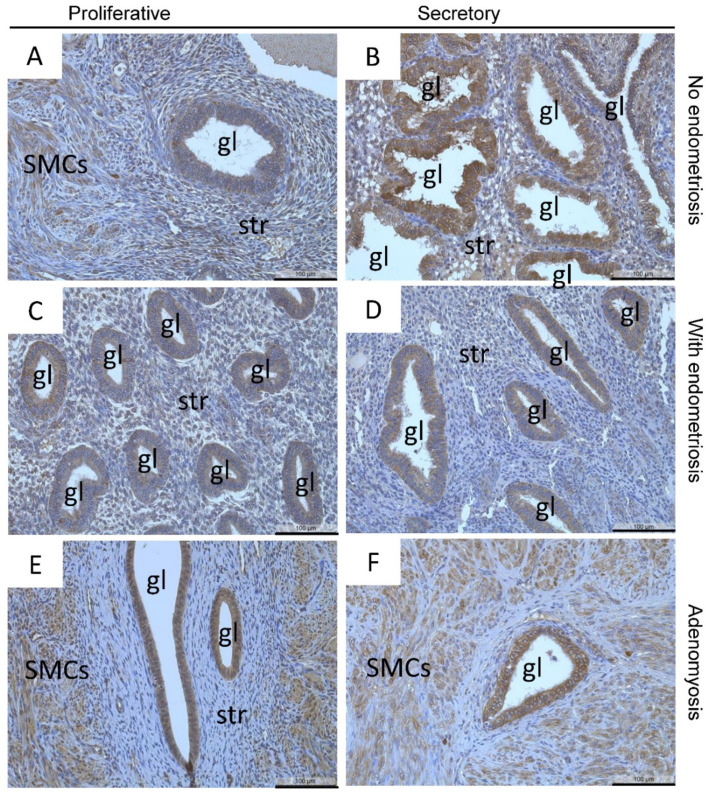
Localization of MT2-MMP was found in the glandular epithelial cells and stromal cells in proliferative (**A**) and secretory (**B**) endometrium of patients without endometriosis and in proliferative (**C**) and secretory (**D**) endometrium of patients with endometriosis as well as in proliferative and secretory adenomyosis (**E**,**F**), respectively. Counterstaining was performed with hematoxylin. Magnification (**A**–**F**) 20×, scale bars 100 µm; gl, gland; str, stroma; SMCs, smooth muscle cells.

Localization of MT2-MMP in patients with and without endometriosis showed low to moderate staining intensity in most glandular epithelial cells and stromal cells in the proliferative and secretory phases (Figure 2A–D) as well as smooth muscle cells in the myometrium (Figure 2E,F), blood vessels, and luminal epithelial cells (data not shown). MT2-MMP was also localized in the proliferative and secretory phase of glandular epithelial cells of adenomyotic patients (Figure 2E,F). Quantification of MT2-MMP localization by HSCORE and percentage of MT2-MMP-positive glands showed no significant differences between the groups in both the proliferative and secretory phase (Table 3 and Table 4). There were also no differences between the eutopic endometrium of patients with or without endometriosis, and therefore, we merged the dataset for further comparison. 

**Figure 3:**
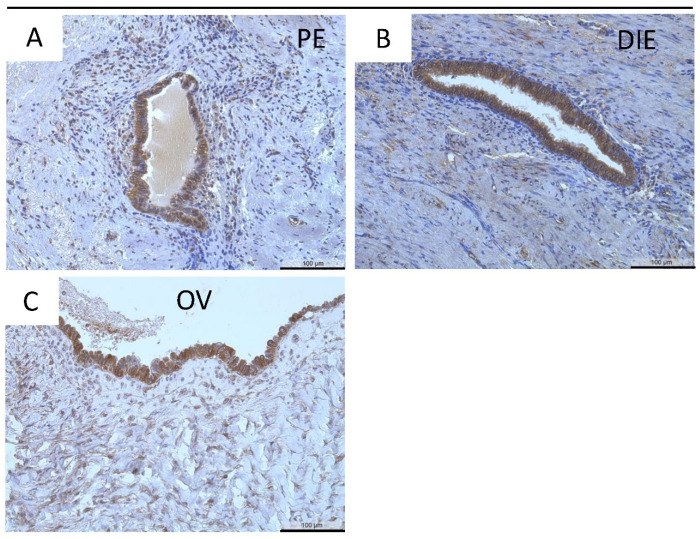
Immunohistochemical detection of MT2-MMP in peritoneal (PE, (**A**)), deep infiltrating (DIE, (**B**)), and ovarian endometriosis (OV, (**C**)). Counterstaining was performed with hematoxylin. Magnification (**A**–**C**) 20×, scale bars 100 µm.

MT2-MMP was further localized in the three endometriotic entities: peritoneal (Figure 3A) deep infiltrating (Figure 3B), and ovarian (Figure 3C) endometriosis. Quantification revealed a significantly lower MT2-MMP HSCORE in ovarian (*p* < 0.001), peritoneal (*p* < 0.01), and deep infiltrating endometriosis (*p* ≤ 0.05) compared to the eutopic endometrium (Figure 2 and Figure 3; Table 5). Furthermore, the percentage of ectopic positive MT2-MMP glands was also significantly lower in the three different endometriotic entities compared to the eutopic endometrium (Table 6). 

**Figure 4:**
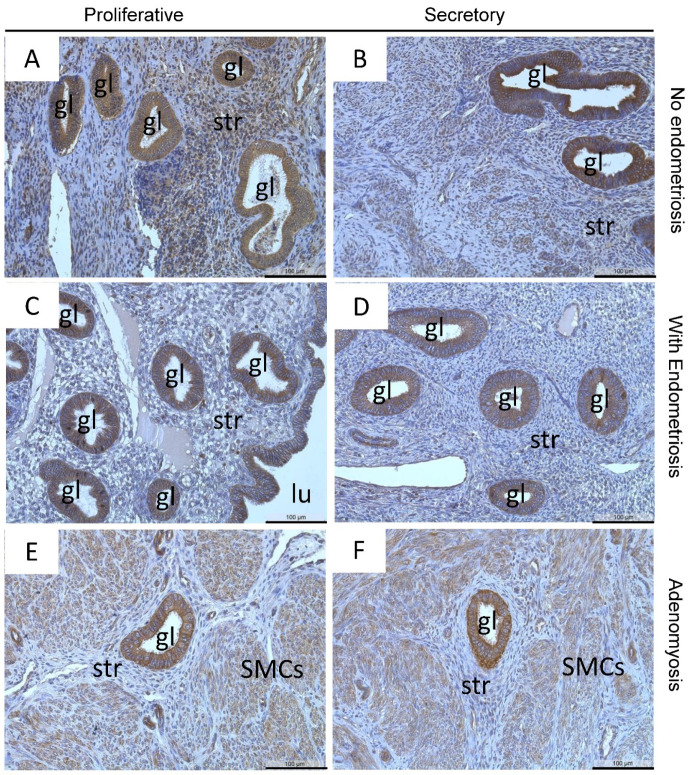
Localization of MT3-MMP was found in the glandular epithelial cells and stromal cells in proliferative (**A**) and secretory (**B**) endometrium of patients without endometriosis, in proliferative (**C**) and secretory (**D**) endometrium of patients with endometriosis, as well as in proliferative and secretory adenomyosis (**E**,**F**), respectively. Counterstaining was performed with hematoxylin. Magnification (**A**–**F**) 20×, scale bars 100 µm; gl, gland; str, stroma; lu, lumen; SMCs, smooth muscle cells.

Endometrial glandular epithelial cells showed strong staining of MT3-MMP in a very high percentage of glands across the menstrual cycle in eutopic endometrium of patients with and without endometriosis (Figure 4A–D). Nearly all luminal epithelial (Figure 4C) but only some stromal cells (Figure 4A–D) are also stained. In adenomyotic lesions MT3-MMP was similarly strongly expressed in the glandular epithelial cells across the cycle in contrast to stromal cells (Figure 4E,F).

The HSCORE of MT3-MMP was highly similar throughout the menstrual cycle in the controls (Table 3) and in the other three groups (EM-En, EM-Ad, and Ad; Table 3). There was also a very high percentage of MT3-MMP-positive glands (89–100%) with no differences between all groups (Table 4); thus, we merged the dataset for further comparison.

**Figure 5:**
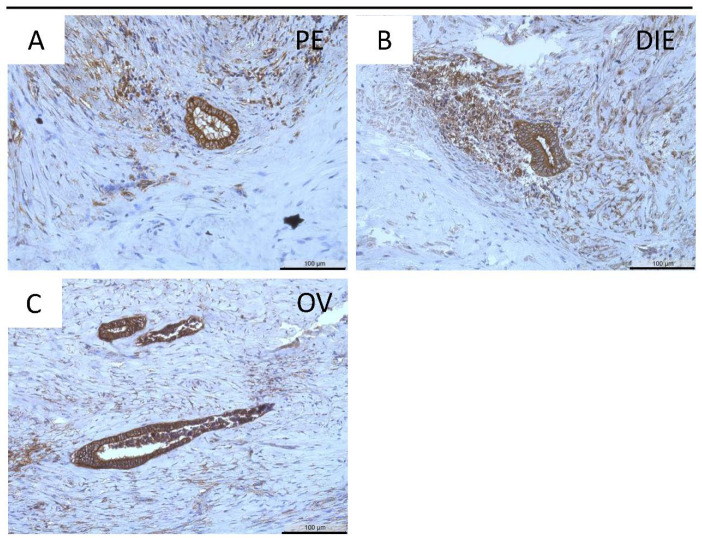
Immunohistochemical detection of MT3-MMP in peritoneal (PE, (**A**)), deep infiltrating (DIE, (**B**)), and ovarian endometriosis (OV, (**C**)). Counterstaining was performed with hematoxylin. Magnification (**A**–**C**) 20×, scale bars 100 µm.

Positive staining for MT3-MMP was further identified in almost all ectopic endometrial epithelial cells: peritoneal (Figure 5A), deep infiltrating (Figure 5B), and ovarian endometriosis (Figure 5C). There was a significantly higher MT3-MMP expression in the eutopic endometrium compared to both peritoneal endometriosis and deep infiltrating endometriosis (*p* < 0.001, Table 5). In contrast, the percentage of MT3-MMP-positive glands was significantly reduced only in deep infiltrating endometriosis compared to eutopic endometrium (*p* ≤ 0.05, Table 6).

## 4. Discussion

In the present study, we investigated MT2-MMP and MT3-MMP expression pattern in eutopic and ectopic endometrial tissues collected from patients with and without endometriosis. Both proteins are mainly found in endometrial epithelial glandular and luminal cells in the eutopic endometrium without cycle dependency. In contrast to MT2-MMP, the HSCORE of MT3-MMP was significantly ~2.5-fold higher (64 ± 3.5 vs. 157 ± 7.0, respectively) and was also found in nearly all glands (65 ± 3.2% vs. 98 ± 1.0%, respectively). The most important finding of the present study is the reduced expression of both proteins in endometriosis but not in adenomyosis and the heterogeneous differences in the three endometriotic entities. 

Membrane type matrix metalloproteinases are involved in tissue breakdown by degradation of ECM proteins and in activation of other MMPs, such as pro-MMP2 [24]. This suggests a possible role of these proteins in establishment and development of adenomyosis by activation of MMP2, which has been shown to be upregulated in patients with adenomyosis [34,35] and endometriosis [36]. Similarly, MT2-MMP is often increased in different types of cancer [37,38,39,40]. Additionally, overexpression of MT2-MMP has been reported to induce proteolysis, leading to epithelial mesenchymal transition (EMT) in carcinomas [41]. MT2-MMP is expressed in several human tissues, such as endothelial cells [42], placenta [43], and leucocytes [44]. MT3-MMP is involved in EMT and neural crest cell migration [45], cell adhesion and lymphatic invasion in melanoma [46], and in human astrocytic tumors [47]. 

In our study, expression and localization of MT2-MMP was observed in human endometrium across the menstrual cycle without cyclical variation, which is in accordance with previous studies [27,28,29]. Furthermore, a preferential expression of MT2-MMP in human endometrial epithelial cells compared to stromal cells and lack of cycle dependency of both proteins was found [27], which is in accordance with our results. In contrast to our findings of a higher expression of MT3-MMP compared to MT2-MMP, they found a similar expression [27]. However, Plaisier et al. [27] only evaluated the staining intensity and not the HSCORE. Furthermore, MT2-MMP protein was also localized within the endothelial cells and smooth muscle cells of the myometrium [27] as found in the present study. In contrast to a previous study showing that the mRNA expression of MT3-MMP is cycle dependent [28], we and Plaisier et al. [27] did not observe any correlation between MT3-MMP expression and phases of the menstrual cycle. With respect to the HSCORE and percentage of positive glands, which were markedly higher for MT3-MMP compared to MT2-MMP, we found nothing in the literature that might explain the differences in the expression level. Nevertheless, it is remarkable that MT3-MMP cleaves more substrates (*n* = 10) compared to MT2-MMP (*n* = 8) and that MT3-MMP interacts with MT1-MMP in contrast to MT2-MMP [24]. 

Interestingly, we identified a reduced MT2-MMP and MT3-MMP protein expression in endometriosis but not in adenomyosis. Furthermore, we also observed a heterogeneous MT2-MMP and MT3-MMP expression among the three distinct endometriotic entities. We recently showed that eutopic endometrial and adenomyotic glands are highly similar with respect to protein abundance of calcyphosine (CAPS) and msh homeobox 1 (MSX1) [19]. Results presented in this study further corroborate our suggestion that endometrial glands are the main source of adenomyotic glands. Furthermore, it might also indicate that endometriosis and adenomyosis do not share a common pathogenesis. In contrast to the tissue breakdown during menstruation, which is the predominant prerequisite for endometriosis, adenomyotic glands seem not to undergo a tissue breakdown. Recently, 3D reconstructions of the human endometrium demonstrated that adenomyotic glands are still connected to the endometrial glands [48], supporting a pathogenesis model of adenomyosis that seems to rely on cellular proliferation and invagination into the myometrium as suggested [16,19]. Therefore, we suggest that the hindrance of cellular migration by Levonorgestrel-releasing intrauterine devices (LNG-IUD) might be best from a cellular viewpoint to inhibit recurrence of endometriosis after laparoscopy, whereas for adenomyosis, inhibitors of cellular invagination need to be identified. Lastly, the heterogeneous expression of MT2/3-MMP in the three different endometriotic entities is in line with our recent hypothesis that most of the differences occur after and not before implantation [49] and might be due to the distinct interactions of the endometrial implants with different microenvironments. 

The current study is based mainly on immunohistochemistry for MT2- and MT3-MMP and thus has some limitations. The mRNA expression of both genes should be investigated with RT-PCR of isolated endometrial and endometriotic stromal and epithelial cells. Furthermore, the different or overlapping functions of both proteins need to be investigated with primary endometrial stromal and epithelial cells and with animal models. 

## 5. Conclusions

Our investigation of the localization of MT2-MMP and MT3-MMP in eutopic and ectopic endometrium in endometriosis and adenomyosis showed that both proteins are localized predominantly in endometrial epithelial glandular and luminal cells in the eutopic endometrium without alterations across the menstrual cycle. The most remarkable finding of the study is the reduced and heterogeneous abundance of both proteins in the three distinct endometriotic entities that is, however, nearly unchanged in adenomyosis. These results further corroborate our recent observations that (1) endometrial glands are the main if not the sole source of adenomyotic glands and (2) that most of the differences of eutopic endometriotic implants occur after and not before implantation, possibly due to the distinct interactions of the endometrial implants with different microenvironments. Furthermore, it may also suggest that endometriosis and adenomyosis do not share a common pathogenesis, which should be further elaborated with suitable in vitro and/or animal models. Further research is needed to understand the functional role of these proteins in eutopic and ectopic endometrium in endometriosis and adenomyosis.

## Figures and Tables

**Table 1:** Overview of the tissue samples used for MT2-MMP.

Tissues	EM	Ov	PE	DIE
All samples	*n* = 56	*n* = 23	*n* = 26 (27)	*n*= 28 (29)
Median age ± SD	36 ± 5.4	36 ± 5.4	33 ± 4.6	32 ± 5.0
Leiomyoma	*n* = 36			
Adenomyosis	*n* = 41			
Adenomyosis only	*n* = 17			
Proliferative	*n* = 25			
Secretory	*n* = 31			
Bladder			3	2
Uterosacral lig			3	3
Ovarian fossa			3	
Pouch of douglas			2	
Round lig of uterus			2	
Pelvic wall			2	
Rectum			2	11
Rectosigmoid				3
Rectovaginal septum			3	5
Paraurethral/rectal			1	1
Sigmoid colon			2	2
Vagina				1
Intestine				1
Scar/Symphysis			2	
Lig latum uteri			1	
Mesovarium			1	

EM, endometrium; Ov, ovarian endometriosis; PE, peritoneal endometriosis; DIE, deep infiltrating endometriosis; SD, standard deviation; lig, ligamentum; *n* = 26 (27), means 27 samples from 26 patients. In contrast to adenomyosis only, adenomyosis also includes patients with endometriosis.

**Table 2:** Overview of the tissue samples used for MT3-MMP.

Tissues	EM	Ov	PE	DIE
All samples	*n* = 47	*n* = 18	*n* = 17 (18)	*n*= 15 (17)
Median age ± SD	43 ± 7.9	35.3 ± 4.0	31 ± 3.7	33 ± 4.6
Leiomyoma	*n* = 30			
Adenomyosis	*n* = 31			
Adenomyosis only	*n* = 13			
Proliferative	*n* = 21			
Secretory	*n* = 26			
Bladder			1	1
Uterosacral lig				3
Ovarian fossa			2	
Pouch of douglas			3	
Round lig of uterus			2	
Pelvic wall			2	
Rectum				4
Rectosigmoid				1
Rectovaginal septum			1	5
Paraurethral/rectal			2	1
Sigmoid colon			1	2
Scar/Symphysis			2	
Lig latum uteri			1	
Mesovarium			1	

EM, endometrium; Ov, ovarian endometriosis; PE, peritoneal endometriosis; DIE, deep infiltrating endometriosis; SD, standard deviation; lig, ligamentum; *n* = 17 (18), means 18 samples from 17 patients. In contrast to adenomyosis only, adenomyosis also includes patients with endometriosis.

**Table 3:** Comparison of MT2/3-MMP in proliferative and secretory phases of endometrium of patients with and without endometriosis and adenomyosis using the HSCORE.

	Ctrl (Healthy)		Em-En		Em-Ad		All	Ad	
MT2-MMP	P	S	P	S	P	S	All	P	S
Mean	67	59	61	56	67	70	64	90	83
SEM	4.6	8.1	8.2	9.3	11.7	6.8	3.5	6.8	5.2
*p*-Value	n.s.	n.s.	n.s.	n.s	n.s.	n.s.	0.0001	n.s.	n.s.
N	6	8	8	10	10	14	56	19	22
Age	35 ± 13	41 ± 5.3	33 ± 6.7	41 ± 6.3	43.5 ± 5.5	45 ± 5.9	42 ± 7.6	44 ± 5.4	45 ± 5.8
MT3-MMP	P	S	P	S	P	S	All	P	S
Mean	191	136	168	128	182	150	157	161	146
SEM	21.6	19.5	21.8	9.5	14	14.6	7.0	13.9	16.3
*p*-Value	n.s.	n.s.	n.s.	n.s.	n.s.	n.s.	0.0001	n.s.	n.s.
N	6	6	6	11	9	9	47	15	16
Age	41 ± 13	43 ± 6.0	32.5 ± 5.8	42 ± 6.0	45 ± 5.0	45 ± 6.8	43 ± 8.0	45 ± 5.1	43.5 ± 6.7

N, number of samples; SEM, standard error of the mean; ctrl, control group (normal endometrium), Em-En, endometrium with endometriosis; Em-Ad, endometrium with adenomyosis; Ad, adenomyosis; P, proliferative; S, secretory; n.s., not significant. The age is given as the median ± standard deviation (SD).

**Table 4:** Comparison of MT2/3-MMP in proliferative and secretory phases of endometrium of patients with and without endometriosis and adenomyosis using the percentage of stained glands.

	Ctrl (Healthy)		Em-En		Em-Ad		All	Ad	
MT2-MMP	P	S	P	S	P	S	All	P	S
Mean	57	65	62	59	70	69	65	84	81
SEM	7.4	7.9	7.2	9.1	9.1	5.7	3.2	5.0	4.6
*p*-Value	n.s.	n.s.	n.s	n.s	n.s.	n.s.	0.0001	n.s	n.s
N	6	8	8	10	10	14	56	19	22
Age	35 ± 13	41 ± 5.3	33 ± 6.7	41 ± 6.3	43.5 ± 5.5	45 ± 5.9	42 ± 7.6	44 ± 5.4	45 ± 5.8
MT3-MMP	P	S	P	S	P	S	All	P	S
Mean	99	89	98	99	100	98	98	95	95
SEM	0.7	7.4	1.9	0.4	0.0	1.2	1.0	3.6	3.2
*p*-Value	n.s.	n.s	n.s.	n.s.	n.s.	n.s.	0.0001	n.s	n.s.
N	6	6	6	11	9	9	47	15	16
Age	41 ± 13	43 ± 6.0	32.5 ± 5.8	42 ± 6.0	45 ± 5.0	45 ± 6.8	43 ± 8.0	45 ± 5.1	43.5 ± 6.7

N, number of samples; SEM, standard error of the mean; ctrl, control group (normal endometrium); Em, endometrium; Em-En, endometrium with endometriosis; Em-Ad, endometrium with adenomyosis; Ad, adenomyosis; P, proliferative; S, secretory; n.s., not significant. The age is given as the median ± standard deviation (SD).

**Table 5:** Comparison of MT2/3-MMP in eutopic and ectopic endometrium using the HSCORE.

	EM (a)	Ov (b)	DIE (c)	PE (d)
MT2-MMP				
Mean	64	33	45	38
SEM	3.5	5.9	5.8	5.9
P	n.s.	a, b < 0.001	a, c ≤ 0.05	a, d < 0.01
N	56	23	29	27
Age	42 ± 7.6	36 ± 5.4	32 ± 5.0	33 ± 4.6
MT3-MMP				
Mean	157	120	101	96
SEM	7.0	5.5	10.1	6.4
P	n.s.	n.s.	a, c < 0.001	a, d < 0.001
N	47	18	17	18
age	43 ± 8.0	35.5 ± 4.4	32 ± 5.2	31 ± 3.7

N, number of lesions; (a–d; groups); EM, eutopic endometrium; Ov, ovarian endometriosis; DIE, deep infiltrating endometriosis; PE, peritoneal endometriosis; SEM, standard error of the mean; n.s., non-significant. For example, a, b < 0.001 means that group a is significantly different from group b. The age is given as the median ± standard deviation (SD).

**Table 6:** Comparison of MT2/3-MMP in eutopic and ectopic endometrium using the percentage of stained glands.

	EM (a)	Ov (b)	DIE (c)	PE (d)
MT2-MMP				
Mean	65	33	42	36
SEM	3.2	5.8	5.1	5.7
P	n.s.	a, b < 0.001	a, c < 0.01	a, d ˂ 0.001
N	56	23	29	27
Age	42 ± 7.6	36 ± 5.4	32 ± 5.0	33 ± 4.6
MT3-MMP				
Mean	98	96	83	85
SEM	1.0	2.6	4.6	4.2
P	n.s. n.s.	n.s. n.s.	a, c ≤ 0.05 b, c ≤ 0.05	n.s. n.s.
N	47	18	17	18
age	43 ± 8.0	35.5 ± 4.4	32 ± 5.2	31 ± 3.7

N, number of lesions; (a–d; groups); EM, eutopic endometrium; Ov, ovarian endometriosis; DIE, deep infiltrating endometriosis; PE, peritoneal endometriosis; SEM, standard error of the mean; n.s., non-significant. For example, a, b < 0.001 means that group a is significantly different from group b. The age is given as the median ± standard deviation (SD).
